# Protease signaling through protease activated receptor 1 mediate nerve activation by mucosal supernatants from irritable bowel syndrome but not from ulcerative colitis patients

**DOI:** 10.1371/journal.pone.0193943

**Published:** 2018-03-12

**Authors:** Sabine Buhner, Hannes Hahne, Kerstin Hartwig, Qin Li, Sheila Vignali, Daniela Ostertag, Chen Meng, Gabriele Hörmannsperger, Breg Braak, Christian Pehl, Thomas Frieling, Giovanni Barbara, Roberto De Giorgio, Ihsan Ekin Demir, Güralp Onur Ceyhan, Florian Zeller, Guy Boeckxstaens, Dirk Haller, Bernhard Kuster, Michael Schemann

**Affiliations:** 1 Human Biology, Technische Universität München, Freising, Germany; 2 Proteomics and Bioanalytics, Technische Universität München, Freising, Germany; 3 Department of Physiology, Shangdong University, Shangdong, China; 4 Nutrition and Immunology, Technische Universität München, Freising, Germany; 5 Department of Gastroenterology and Hepatology, Academic Medical Center, Amsterdam, The Netherlands; 6 Medical Clinic, Academic Hospital, Vilsbiburg, Germany; 7 Medical Clinc II, Helios Hospital, Krefeld, Germany; 8 Department of Medical and Surgical Sciences, St. Orsola Hospital, Bologna, Italy; 9 Department of Clinical Sciences, Nuovo Arcispedale S. Anna, University of Ferrara, Ferrara, Italy; 10 Department of General Surgery, University Hospital Rechts der Isar, Technische Universität München, Germany; 11 Surgery, Academic Hospital, Freising, Germany; 12 Translational Research Centre for Gastrointestinal Disorders, University Hospital Gasthuisberg, Catholic University of Leuven, Leuven, Belgium; University of California Los Angeles, UNITED STATES

## Abstract

**Background & aims:**

The causes of gastrointestinal complaints in irritable bowel syndrome (IBS) remain poorly understood. Altered nerve function has emerged as an important pathogenic factor as IBS mucosal biopsy supernatants consistently activate enteric and sensory neurons. We investigated the neurally active molecular components of such supernatants from patients with IBS and quiescent ulcerative colitis (UC).

**Method:**

Effects of supernatants from 7 healthy controls (HC), 20 IBS and 12 UC patients on human and guinea pig submucous neurons were studied with neuroimaging techniques. We identify differentially expressed proteins with proteome analysis.

**Results:**

Nerve activation by IBS supernatants was prevented by the protease activated receptor 1 (PAR1) antagonist SCHE79797. UC supernatants also activated enteric neurons through protease dependent mechanisms but without PAR1 involvement. Proteome analysis of the supernatants identified 204 proteins, among them 17 proteases as differentially expressed between IBS, UC and HC. Of those the four proteases elastase 3a, chymotrypsin C, proteasome subunit type beta-2 and an unspecified isoform of complement C3 were significantly more abundant in IBS compared to HC and UC supernatants. Of eight proteases, which were upregulated in IBS, the combination of elastase 3a, cathepsin L and proteasome alpha subunit-4 showed the highest prediction accuracy of 98% to discriminate between IBS and HC groups. Elastase synergistically potentiated the effects of histamine and serotonin–the two other main neuroactive substances in the IBS supernatants. A serine protease inhibitor isolated from the probiotic *Bifidobacterium longum* NCC2705 (SERPIN_BL_), known to inhibit elastase-like proteases, prevented nerve activation by IBS supernatants.

**Conclusion:**

Proteases in IBS and UC supernatants were responsible for nerve activation. Our data demonstrate that proteases, particularly those signalling through neuronal PAR1, are biomarker candidates for IBS, and protease profiling may be used to characterise IBS.

## Introduction

The enteric nervous system (ENS) in the gut wall coordinates and maintains normal gut functions. Its central role for normal motility, secretion and immune cell function also indicates that altered ENS function is often associated with gut pathologies. Irritable bowel syndrome (IBS) is a functional gastrointestinal disease and belongs with a population prevalence of ~11% to the most common gut disorders [1]. The heterogeneity of IBS phenotypes, usually defined by their stool irregularities, makes it difficult to pinpoint a specific cause [1]. One emerging pathophysiological concept is an altered signaling along a mucosa-immune-nerve axis which includes the brain-gut- axis. This pathway integrates signals arising from the gut lumen as well as the microenvironment in the gut wall, and it thus responds not only to physiological stimuli but also to potentially harmful challenges. The use of mucosal biopsies and biopsy supernatants became a powerful tool to investigate gut pathologies and to link altered cellular behavior with gut dysfunctions [2]. Evidence for this is that mucosal biopsy supernatants from IBS patients, but not from healthy controls, activate enteric neurons [3], vagal afferents [4] as well as spinal afferents [5]. Strikingly, this excitatory action is a consistent feature of IBS supernatants independent of whether tested in human or guinea pig enteric neurons and occurs independent of IBS phenotypes [3,5,6]. However, the degree of neural activation is associated with abdominal pain scores [5]. Proteases contribute most as the broad serine protease inhibitor FUT-175 prevented supernatant evoked nerve activation [3]. Moreover, proteases are responsible for the synergistic potentiation of the other neuroactive components in the supernatant, namely histamine and serotonin [7]. Notably, actions of serine proteases on human enteric neurons are strongly dependent on protease activated receptor (PAR) 1 rather than PAR2 or PAR3 [8,9]. This agrees with the finding that the potentiating effect of tryptase on histamine and serotonin responses is not PAR2 mediated [7]. Proteases are not only responsible for acute nerve activation but also account for desensitization of nerve responses. This explains why a mediator cocktail containing the neuroactive components of the IBS biopsy supernatants evokes less activation of enteric neurons in biopsies from IBS patients compared to healthy controls [10].

Furthermore, protease actions may be also relevant in inflammatory bowel diseases, in particular in symptom generation [11]. IBS-like symptoms are common in patients with quiescent ulcerative colitis (UC) which has led to the, admittedly controversial, hypothesis that IBS and UC share some common pathological factors [12–14]. For example, the mucosa of IBS and UC patients shows comparable deficits in serotonergic signaling pathways [15] as well as defects in epithelial barrier function [16]. Additionally, the mucosa of IBS patients shows signs of low grade inflammation including increased number and/or reactivity of immune cells as well as increased levels of pro-inflammatory cytokines [17].

Despite numerous efforts, the heterogeneity of patients has so far hampered research aimed at identifying reliable biomarkers for IBS. Thus, the focus should be to identify some eligible markers and treatable traits which help to better define and treat at least a subpopulation of IBS patients. As outlined above, proteases and their signaling pathways seem to be attractive candidates [18].

This study aimed to support the relevance of proteases and in particular to highlight the role of PAR1 in IBS. Furthermore, we wanted to provide the basics for the development of protease-based biomarkers for IBS.

We succeeded to demonstrate the importance of PAR1 for biopsy supernatant induced nerve activation in IBS but not UC. In addition, we found a protease expression pattern which distinguished IBS from healthy controls and UC patients in remission. Finally, we provided evidence that a serine protease inhibitor from the probiotic strain *Bifidobacterium longum* (SERPIN_Bl_) was able to prevent the nerve activation by IBS mucosal supernatants.

## Methods

### Study participants and tissue samples

All protocols and procedures performed on human subjects and samples were approved by the appropriate Ethics Committees (TU München: Prot.No 906/2006 and 1925/2004; Academic Medical Center Amsterdam: trial register NTR39, ISRCTN22504486; University Bologna: Prot.No. 64–2004, Clinic Krefeld (academic hospital of University Düsseldorf, approval number 3166) and conformed to the standards set by the *Declaration of Helsinki*. Informed written consent was obtained from all subjects. Characteristics of study participants, sample collection and studies with samples are summarized in S1 Table. IBS patients were diagnosed according to Rome II or III criteria and had symptoms at the time of endoscopy. The IBD patients had histologically confirmed UC and their biopsy samples were taken during a phase of endoscopically confirmed clinical remission. Patients were allowed to keep their maintenance treatment up to 24hrs before endoscopy. Healthy controls (HC) had no abdominal symptoms, no allergic disorder and no organic disease. During the endoscopy, 4 biopsies were collected from the descending colon and rapidly immersed in either 1 ml carbogen-aerated (95% O2/5% CO2) 37°C warm Hank’s solution (n = 13), (Sigma, St. Louis, MO) or in 1 ml oxygenated (100% O2 or ambient air) 37°C warm HEPES/Krebs buffer (pH 7.4) containing in mM: 135 NaCl, 5.4 KCl, 1 MgCl2·6H2O, 1.2 NaH2PO4, 3 HEPES, 1.25 CaCl2·2H2O and 12.5 glucose (all from Sigma, Steinheim, Germany) (n = 26). After an incubation of 25 min, the supernatants were centrifuged at 200–4000g (for HEPES/Krebs supernatants Spin-X micro tubes (Corning Life Science) were used), aliquoted and stored at -70°C until use. The more recent samples were incubated in HEPES/Krebs buffer because ambient air is sufficient for its oxygenation and pH control. This turned out as a substantial practical benefit for the sample collection in medical units. The effects of the supernatants on the neurons were not affected by the use of different buffers. The neuroindex evoked by IBS supernatants incubated in Hank´s (n = 8, 270 (218./429)) or in HEPES/Krebs buffer (n = 6, 240 (164/372) did not differ (*P* = 0.573, Mann-Whitney Rank Sum Test).

All guinea-pig work was conducted according to the German guidelines for animal care and welfare (Deutsches Tierschutzgesetz, revised according to the Directive 2010/63/EU) and approved by the Bavarian state ethics committee (Regierung Oberbayern, which serves as the Institutional Care and Use Committee for the Technische Universität München) according to §4 and §11 Deutsches Tierschutzgesetz under the reference number 32-568-2. Male guinea pigs (Dunkin Hartley (280-400g), Harlan GmbH, Borchen, Germany) were killed by cervical dislocation followed by exsanguination.

### Neuroimaging of supernatants effects on human and guinea pig enteric neurons

Macroscopically normal intestinal specimens (as determined by visual inspection by a pathologist) were taken from 105 patients (51 male, 54 female, mean age 69) who underwent surgery for colon carcinoma/adenoma (60/4), diverticular disease (15), pancreas carcinoma (8), stenosis (4), polyps (4), gastric or mesenteric carcinoma (3), stoma reversal (3) and 1 each for small intestinal carcinoma, pancreatisis, polytrauma or neuroendocrine tumor. Effects of the supernatants were tested either in human or in guinea-pig submucous plexus preparations as previously described [3,5]. Briefly, ganglion neurons were stained with the fluorescent voltage-sensitive dye 1-(3-sulfonatopropyl)-4-[β[2-(di-n-octylamino)-6-naphthyl]vinyl]-pyridinium betaine (Di-8-ANEPPS 20 μM; Molecular Probes Mobitec, Göttingen, Germany). Spike discharge was recorded from Di-8-ANEPPS stained neurons by an array of 464 photodiodes (RedShirt Imaging, Decatour, GA, USA) with a 40x objective (UAPO/340 Olympus, Hamburg, Germany) at a sample frequency of 1.6 kHz. Representative responses to pressure application of drugs or mucosal biopsy supernatants (diluted 1:1 with Krebs buffer) were recorded with 1.3-5s recording periods. The PAR1 antagonist SCH79797 (10μM, Tocris Bioscience/R&D Systems; Bristol UK) was added to the superfusing Krebs solution. We previously validated specificity as well as the concentration of SCH79797 in our samples [9]. In some experiments, supernatants were incubated before application with the broad-spectrum serine protease inhibitor FUT-175 (50 g/mL; Calbiochem, Darmstadt, Germany).

We previously reported that in mucosal biopsy supernatants from IBS patients two non-protease neuroactive components, serotonin and histamine, may interact in a synergistic manner with proteases to evoke neuronal activation (Ostertag et al., 2017). To follow up this idea we studied synergisms between elastase (324681, Merck KGaA, Darmstadt, Germany) and serotonin and histamine (both from Sigma Aldrich, Steinheim, Germany). For these studies we used calcium-imaging as previously described in detail [7]. We used Fluo-4 AM (Invitrogen, Darmstadt, Germany) to monitor changes in intracellular calcium levels ([Ca^2+]^_i_). In the present study, the effects of a 600ms the effects of a combination of 1μmol/l histamine and 1μmol/l serotonin with and without 100nmol/l human elastase on human colonic submucous neurons were compared. The concentrations of serotonin and histamine were based on those measured in IBS supernatants [3] taking into account that with our pressurized micro ejection system there is a 1:10 dilution before a substance reaches the ganglion [19].

The serine protease inhibitor from *Bifidobacterium longum* NCC2705 (SERPIN_BL_), which efficiently inhibits elastase activity, was produced using genetically engineered *Escherichia coli* overexpressing SERPIN_BL_ (kindly supplied by Nestle Research Center, Lausanne, CH) [20]. *Escherichia coli* SERPIN_BL_ was grown in LB + ampicillin (100 μg/ml, Sigma Aldrich, Steinheim, Germany) (37°C, aerobe, shaker) until an OD600 of 0.6 was reached. Isopropyl-β-D-thiogalactopyranosid 0.1 mM) was added (37°C, aerobe, and shaker, overnight). Bacterial cells were harvested (4500 g, 10 min) and resuspended in lysis buffer (1xLEW buffer included in Macherey-Nagel Kit for purification of His-tagged proteins (1 mg/ml lysozyme, 1 mM PMSF; less than 5% of the original culture volume, Macherey Nagel, Düren, Germany). The bacterial suspension was placed on ice, sonicated (3x15 strokes, amplitude 75, and cycle 0.5) and bacterial debris was pelleted (10000 g, 15 min). His-tagged SERPIN_BL_ in the supernatant was purified according to the manufacturers´ instructions using Ni-TED 2000-columns (Macherey Nagel, Düren, Germany). The elution buffer was exchanged to Krebs buffer using concentration columns (exclusion membrane 10 kDa, 4°C) (Sartorius, Göttingen, Germany). Mucosal biopsy supernatants were incubated for 10min with 15nM SERPIN_BL_ before application to submucous neurons.

### Analysis of neuroimaging data

Analysis of imaging experiments was performed with Neuroplex 10.1.2. (RedShirtImaging, Decatur, GA, USA) as described before [21]. To analyze the proportions of enteric neurons responding to the supernatants we counted the Di-8-ANEPPS labeled neurons per ganglion. Individual neurons can be visualized since the dye incorporates into the membrane revealing the outline of individual cell bodies. The overlay of signals with the ganglion image allowed us to analyze the response of individual neurons. As parameters for the supernatant evoked activation of submucous neurons we calculated the neuroindex per ganglion which is the product of the percentages of responding neurons and their average spike discharge frequency [Hz]. Throughout the manuscript the neuroindex values are presented without units. Numbers of tissues (equivalent to number of patients), ganglia, and neurons are given in sequence without further specification, e.g. a result based on experiments from 4 tissues, 5 ganglia, and 20 neurons is presented as (4/5/20). For the calcium-imaging experiments the maximal intracellular calcium increase [Ca^++^]_I_ relative to resting light level (ΔF/F) was determined for each application. Here the number of nicotine responsive cells was taken as 100% for the calculation of the percentage of neurons responding to individual substances or a mixture of substances with a [Ca^++^]i signal [7]. The parameter to determine neuronal activation was the peak [Ca^++^]i response for each neuron expressed as ΔF/F. To determine a value for the overall activity in each ganglion, we multiplied the mean peak [Ca^++^]i response with the percentage of responding neurons per ganglion to obtain a Ca-neuroindex.

Data are expressed as the median with the 25^th^ and 75^th^ quartiles (in brackets). Statistical tests and graphs were performed using SigmaPlot 12.5 (Systat Software Inc., Erkrath, Germany). Differences between the nerve activating effects of supernatants from HC, IBS and UC patients were tested with the Kruskal-Wallis one way analysis of variance on ranks combined with a post-hoc all pairwise multiple comparison test (Dunn´s method). A Bonferroni procedure was applied. Effects of mediator combinations were compared in an unpaired fashion using the Mann-Whitney Rank Sum Test. The effects of the FUT-175, the PAR1 antagonist and SERPIN_BL_ were tested in a paired experimental design. Thus the Wilcoxon signed rank test for paired data was used for statistical comparison. For all tests a *P* value of <0.05 was considered significant.

### Proteome analysis of the supernatants

For some supernatants a proteome analysis was performed as previously described [22]. Samples were run into a 4–12% NuPAGE gel (Invitrogen, Darmstadt, Germany) for about 0.5 cm to desalt and concentrate the sample prior to in-gel tryptic digestion, which was performed according to standard procedures.

Nanoflow liquid chromatography tandem mass spectrometry (LC-MS/MS) was performed by coupling an Eksigent nanoLC-Ultra 1D+ (Eksigent, Dublin, CA) to an Orbitrap Elite mass spectrometer (Thermo Scientific, Bremen, Germany). Peptides were delivered to a trap column (100 μm × 2 cm, packed in-house with Reprosil-Pur C_18_-AQ 5 μm resin; Dr. Maisch, Ammerbuch, Germany) at a flow rate of 5 μL/min in 100% solvent A (0.1% formic acid in HPLC-grade water). After 10 min of loading and washing, peptides were transferred to an analytical column (75 μm × 40 cm, packed in-house with Reprosil-Pur C_18_-AQ, 3 μm resin; Dr. Maisch, Ammerbuch, Germany) and separated via a 210 min gradient from 7% to 35% solvent B (0.1% formic acid in acetonitrile) at 300 nL/min flow rate.

The mass spectrometer was operated in data-dependent mode, automatically switching between MS and MS2. Full-scan MS spectra were acquired in the Orbitrap at 30 000 (m/z 400) resolution after accumulation to a target value of 1 000 000. Internal calibration was performed by use of the ion signal [Si(CH_3_)_2_O]_6_H^+^ at m/z 445.120 025 present in ambient laboratory air. Tandem mass spectra were generated for up to 15 peptide precursors for fragment by using higher energy collisional dissociation (HCD) at normalized collision energy of 30% and a resolution of 15 000 with a target value of 100 000 charges after accumulation for a maximum of 100 ms.

Quantitative analysis with intensity-based label-free quantification was performed within the Progenesis software (version 3.1, Nonlinear Dynamics, Newcastle, U.K.). MS/MS spectra were transformed into peak lists and exported as Mascot generic files. The Mascot generic files were searched against the UniProtKB ‘complete proteome’ sequence database (download date 26 Oct 2010; 110,550 sequences) by use of Mascot (version 2.3.0, Matrix Science, London). Search parameters were as follows: precursor tolerance 10 ppm, fragment tolerance 0.02 Da, full tryptic specificity with up to two missed cleavage sites, mis-assignment of the monoisotopic peak to the first ^13^C peak, variable modification of carbamidomethylation of cysteine residues and methionine oxidation. Search results for spectrum to peptide matches were exported in.xml format and then imported into Progenesis software to enable the combination of peptide quantification and identification. Peptides with Mascot ion scores <31 (*p* = 0.05 identity threshold) were discarded, and only unique peptides for corresponding proteins were used for identification and quantification.

### Statistical analysis of proteome data

Statistical analysis of quantified proteins was performed using R (version 3.1.2). Raw protein abundance values were normalized using Variance Stabilization Normalization (VSN). The VSN normalization has been employed to ensure that differences in the sample amount are normalized on the level of protein intensity distributions [23]. Differential expression was assessed with a moderated linear model using the limma package in Bioconductor. Differences in protein expression were estimated with the least squares linear model fitting procedure and tested for differential expression with moderated F-statistic via the empirical Bayesian statistics described in the limma package. We accepted or rejected the null hypothesis on the basis of *P* values computed via limma at a specified significance level. *P* values were adjusted for multiple testing to control the false discover rate at 5%. For multiple testing adjustments, we calculated the false discover rate using the algorithm of Benjamini and Hochberg. *P* values, with appropriate multiple testing adjustment to control the false discovery rate at 5% allowed us to identify differentially expressed proteins. Cluster analyses including principal component analysis (PCA) and hierarchical clustering were performed using standard algorithms and metrics. Hierarchical clustering was performed using complete linkage with Euclidean metric.

To further assess how well a single or a combination of proteins differentially expressed in the three study groups (HC, IBS and UC) can classify the individual supernatant sample into the right group, we performed a linear discriminant analysis (LDA) to construct a predictive model. The performance of the model is indicated by accuracy of prediction, which is defined as proportion of correct predictions. To avoid overfitting, the samples were randomly divided into training and testing sets. The training sets were used to fit the model, and then the fitted model was used to predict the classification of remaining samples. 100 replicates were done for each of the combination of predictors. A receiver operating characteristic (ROC) analysis was performed demonstrating how well a classifier (e.g. a particular protein) can correctly classify the individual supernatant samples into two different groups e.g. UC and IBS patient groups. A measure of the classification accuracy of a selected classifier is the area under the curve (AUC) of the ROC plots. An AUC value of 0.5 refers to a random classifier, a value of 1.0 represents a perfect classifier.

## Results

### Effects of IBS and UC supernatants on submucous neurons

**Fig 1:**
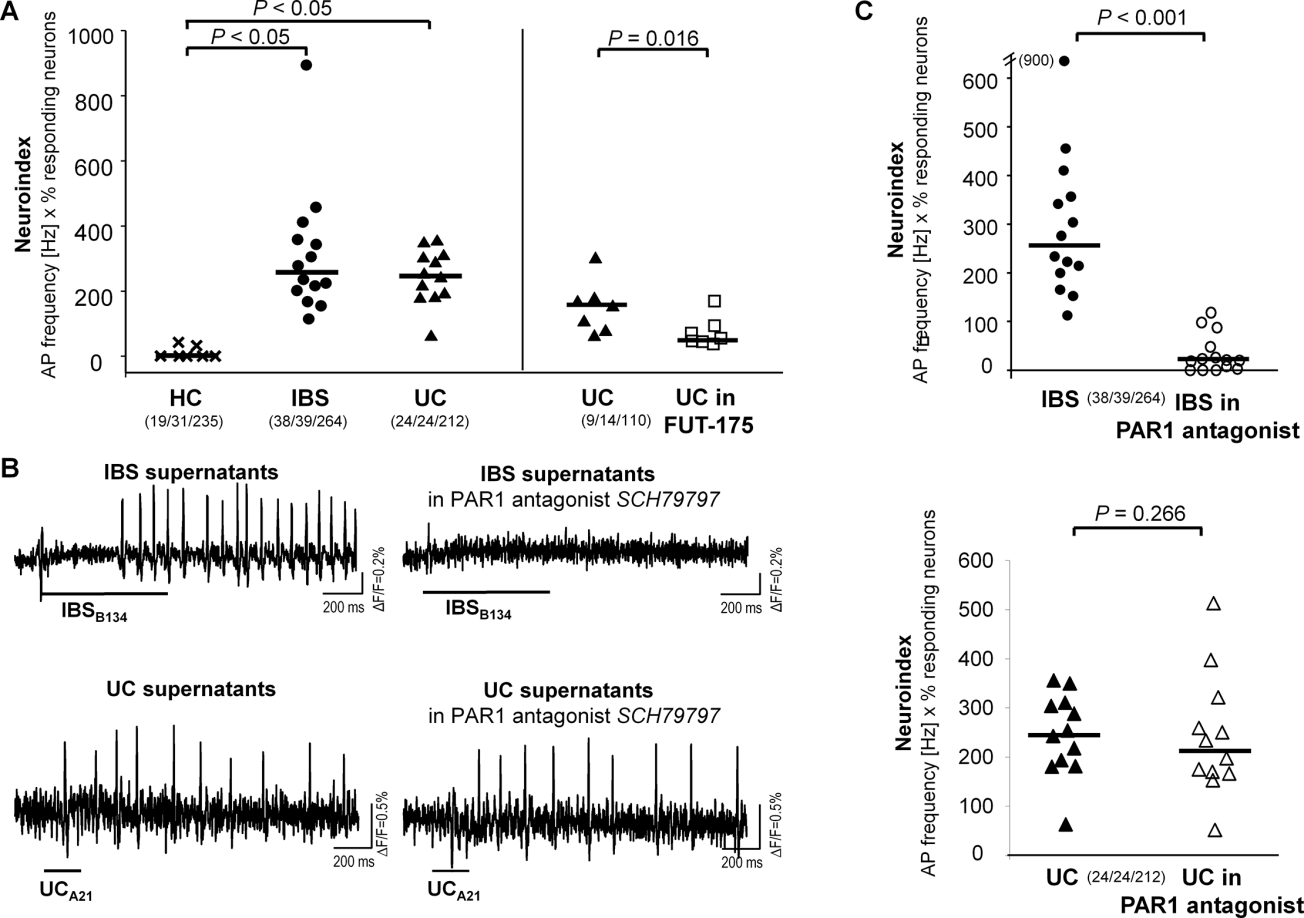
Nerve activation evoked by mucosal biopsy supernatants. (A, left panel) Mucosal biopsy supernatants from patients with irritable bowel syndrome (14 IBS) and patients with ulcerative colitis in remission (12 UC), but not from healthy controls (7 HC), caused nerve activation as indicated by a significantly increased neuroindex (product of spike frequency and % of responding neurons) (Dunn´s method). (A, right panel) the broad spectrum serine protease inhibitor FUT-175 reduced the neuroindex evoked by UC supernatants (Wilcoxon signed rank test for paired data). (B) Spike discharge in human submucous neurons after pressure application of supernatants (duration indicated by the bars below the traces) from IBS (upper panel) and UC patients (lower panel) before and during incubation with the PAR1 receptor antagonist SCH79797 (10μM). While SCH79797 blocked spike discharge in response to IBS supernatants, it had no effect on spike discharge after application of UC supernatants (each symbol represent one patient sample). (C) Quantification of the neuronal activity evoked by biopsy supernatants. The PAR1 receptor antagonist SCH79797 significantly reduced the neuroindex evoked by IBS supernatants from 14 patients (upper panel) but had no influence on the neuronal activation evoked by UC supernatants from 12 patients (lower panel) (each symbol represent one supernatant; Wilcoxon signed rank test for paired data). Numbers in parentheses indicate number of tissues/ganglia/neurons studied.

Spritz application of HC supernatants had only marginal effects on spike discharge in human submucous neurons (Fig 1A). In contrast, IBS and UC supernatants evoked a strong activation (Fig 1A and 1B). IBS and UC supernatants evoked similar spike discharge frequencies (IBS: 3.8 Hz (2.5/5.5 Hz); UC: 3.3 Hz (2.2/3.8 Hz) in similar proportions of neurons (IBS: 81% (52/95%); UC: 80% (71/83%). Accordingly, the neuroindex as the product of spike frequency and proportion of responding neurons was similar with UC and IBS supernatants (Fig 1A).

We previously reported that the serine protease inhibitor FUT-175 blocked nerve activation evoked by IBS supernatants in human and guinea pig submucous plexus preparations [3,5,6]. In the present study, FUT-175 significantly decreased effects of UC supernatants from 7 patients without fully preventing spike discharge (Fig 1A); the neuroindex before and after FUT-175 incubation was 158.3 (83.1/182.5) and 50.5 (39.5/89.0), respectively (9/14/110, P = 0.016). Because PAR1 was mainly involved in protease induced activation in human ENS [9], we perfused the PAR1 antagonist SCH79797. This significantly inhibited the nerve activation by IBS supernatants but the response to UC supernatants remained unchanged (Fig 1B and 1C).

### Proteome analysis of the supernatants

**Fig 2:**
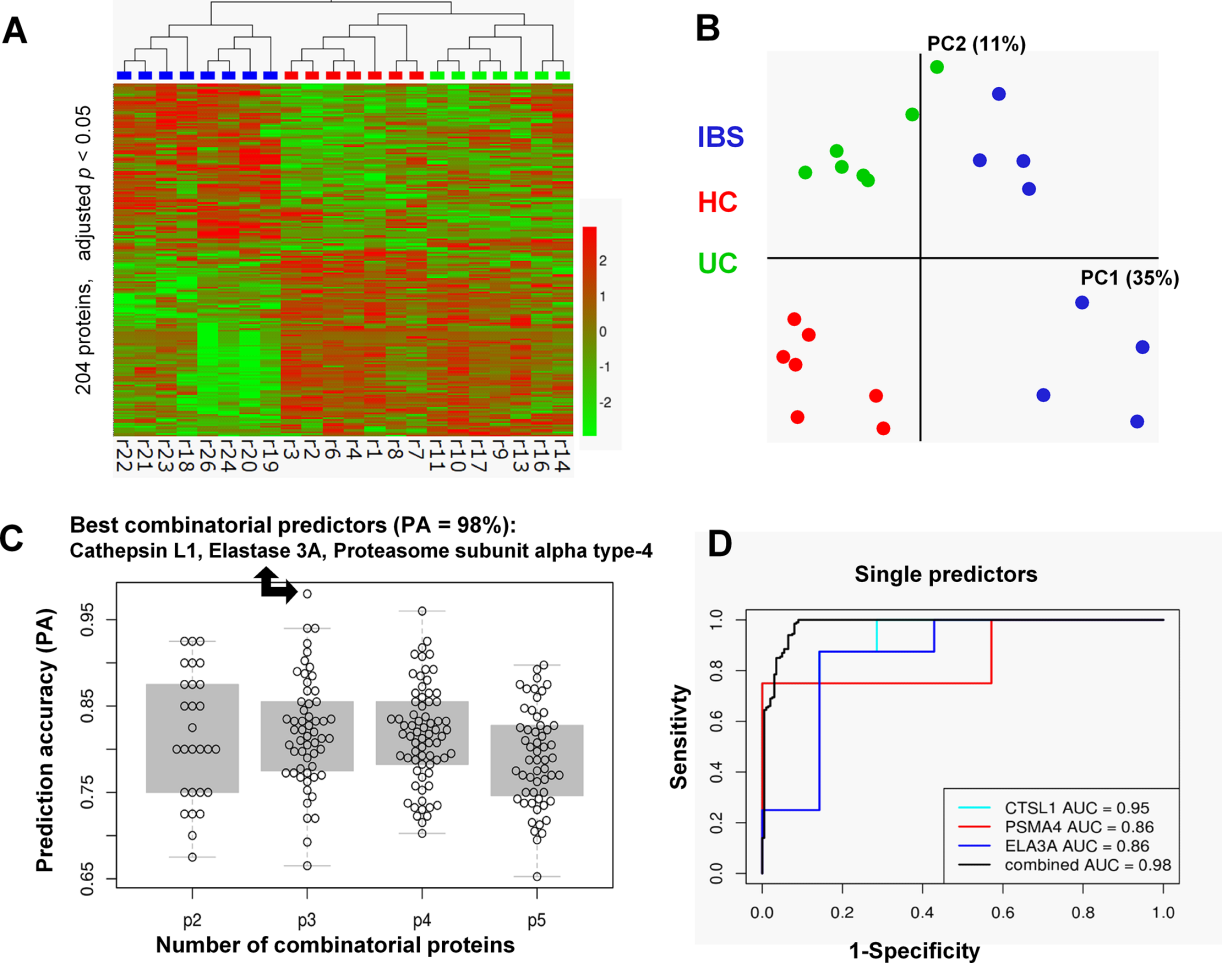
Proteome analysis of mucosal biopsy supernatants. Proteome analysis revealed significantly different protein levels and pattern in biopsy supernatants from IBS, HC or UC. (A) 204 proteins exhibit different levels between the three groups (*p* < 0.05, Benjamini-Hochberg adjusted). Hierarchical clustering of z-transformed protein levels reveals striking differences between biopsy supernatants. (B) Principal component analysis of all 22 samples using the 204 proteins corroborates this finding. (C) The subset of the 8 proteins which were significantly upregulated in the IBS patients vs. healthy controls were used to construct predictive models using linear discriminant analysis (left panel). All combinations consisting of 2 to 5 proteins (p1-p5) were examined. The prediction accuracy of each is shown as a dot in the figure. The combination of the three proteases cathepsin L1 (CTSL1), proteasome subunit alpha type 4 (PSMA4), and elastase 3A (ELA3A) resulted in the highest value of prediction accuracy of 98%, marked with an arrow. Single ROC curves plotted for the three protein predictors CTSL1, PSMA4, and ELA3A are shown in the right panel. The area under the curve (AUC), as a measure of the discriminatory value of individual or combinatorial proteins, showed the highest value for the protein combination. Note, for none of the individual proteins the specificity to discriminate between the HC and the IBS groups was lower than 85% at a given sensitivity of 75%.

A proteome analysis of the supernatants identified 204 out of 1081 proteins as differentially expressed between IBS, UC and HC (Fig 2, see S2 Table for all 1081 proteins), thereby revealing different proteome profiles (Fig 2A and 2B). Strikingly, the difference in proteome profile of supernatants was more pronounced between IBS and HC than between UC and HC (compare columns in heat map in Fig 2A). Considering our findings on supernatant evoked nerve activation, we primarily focused further analyses on differentially expressed proteases. Altogether 17 proteases were differentially expressed in IBS, HC and UC supernatants (Table 1). Of those, 4 proteases, namely elastase 3a, chymotrypsin C, proteasome subunit type beta-2 and an unspecified isoform of complement C3, were significantly more abundant in IBS compared to HC and UC supernatants. Additional 4 proteases were more and 6 less abundant in IBS compared to HC supernatants.

**Table 1:** Differentially expressed proteases in the biopsy supernatants of patients with IBS, UC, and controls.

				IBS vs. HC		UC vs. HC		IBS vs. UC	
UniProt ID_HUMAN	Name	Localization	Function	log_10_ ratio	*P* value	log_10_ ratio	*P* value	log_10_ ratio	*P* value
1. Q96QL8	Elastase 3A	Extracellular	Serine type endopeptidase with some elastolytic action, digestive function, intestinal cholesterol metabolism	0.63	0.026	0.22	0.870	0.41	0.025
2. A8MTQ9	Chymotrypsin-C	Extracellular	Serine type endopeptidase (pancreatic), some elastolytic action, chymotrypsin-like function, activation and degradation of trypsinogens and procarboxypeptidases	0.60	0.014	0.21	0.977	0.39	0.003
3. D3DPS0	Proteasome subunit beta type-2	Cytoplasm	Multicatalytic threonine peptidase, caspase-,trypsin-, and chymotryosin-like activity, antigen processing and presentation via MHC class I	0.38	0.006	0.10	0.500	0.28	0.034
4. CO3	Complement C3 (unspecified isoform)	Extracellular	Molecule system including one/several serine proteases, activation of complement system, antimicrobial action.	0.29	0.016	-0.15	0.366	0.44	0.002
5. B4DJQ8	Cathepsin C	Cytoplasm	Cysteine endopeptidase controlling activation of serine proteases including elastase in inflammatory cells	1.18	0.001	1.10	0.007	0.07	0.493
6. B3KQK4	Cathepsin L1	Cytoplasm	Cysteine endopeptidase with collagen and elastase as substrates; control of neutrophil elastase activity	0.51	0.005	0.29	0.309	0.22	0.081
7. DNPEP	Aspartyl aminopeptidase	Cytoplasm	Intracellular protein and peptide metabolism	0.37	0.006	0.24	0.219	0.14	0.276
8. D3DW86	Proteasome subunit alpha type-4	Cytoplasm	Multicatalytic threonine peptidase, immune defense, host-virus interaction	0.16	0.017	0.05	0.133	0.11	0.113
9. B2R7F8	Plasminogen	Extracellular	Serine type endopeptidase; cell surface; precursor of plasmin; pro inflammatory; immune defense	0.27	0.450	-1.33	0.073	1.60	0.006
10. CO3	Complement C3	Extracellular	Molecule system including one/several serine proteases, activation of complement system, antimicrobial action.	-0.10	0.823	-1.53	0.014	1.43	0.004
11. Q5U000	Cathepsin Z	Cytoplasm	Cysteine carboxypeptidase with broad specificity, immune processes	-0.55	0.013	-0.22	0.034	-0.33	0.569
12. B2CIS9	Caspase 14	Cytoplasm	Apoptosis-related cysteine peptidase	-0.49	0.006	-0.18	0.091	-0.31	0.086
13. Q6IAT9	Proteasome subunit beta type-6	Cytoplasm	Multicatalytic threonine peptidase, ATP-dependent proteolytic activity, caspase-like activity, immune defense	-0.40	0.019	-0.10	0.107	-0.31	0.221
14. Q5QNR8	Proteasome subunit beta type-8	Cytoplasm	Multicatalytic threonine peptidase, ATP-dependent proteolytic activity, immune defense, facilitates apoptosis	-0.28	0.007	-0.16	0.043	-0.12	0.315
15. A8K071	X-prolyl aminopeptidase 1	Cytoplasm	Metalloaminopeptidase; degradation of tachykinins, neuropeptides, and peptide hormones	-0.62	0.016	-0.28	0.640	-0.35	0.037
16. A8K6H1	Endoplasmic reticulum aminopeptidase 1	Extracellular	Zinc metallopeptidase, ubiquitous, immune defense, presentation of MHC class I presentation	-0.26	0.046	0.15	0.519	-0.41	0.012
17. Q6IBC3	Cathepsin H	Cytoplasm	Cysteine endo-and exopeptidase, enkephalin and galanin neurotransmitter production	-0.31	0.094	0.14	0.338	-0.44	0.020

*P* values for the F-test between all three groups were < 0.036; UniProt ID: universal protein database identification number: log_10_ ratio: differences in the log10 protein abundance; listed are those peptidases showing a significant difference between each of two study group

1–4 higher abundance in IBS versus HC and UC. 5 higher abundance in IBS versus HC and UC versus HC. 6–8 higher abundance only in IBS versus HC. 9–10 higher abundance in IBS versus UC. 11–14 lower abundance in IBS versus HC. 15–16 lower abundance in IBS versus HC and UC. 17 lower abundance in IBS versus UC. Data on function from Uniprot

The distinct nerve activating properties and proteome profiles clearly distinguish IBS from UC and HC supernatants. We performed a linear discriminant analysis with combinations of proteases that distinguish IBS from HC supernatants and used a predictive model to classify IBS and HC samples (Fig 2C). The highest prediction accuracy of 98% was achieved with a combination of elastase 3A, cathepsin L1, and proteasome subunit alpha type 4 (Fig 2). The second and third best fit with an accuracy of 94% were the combinations aspartyl aminopeptidase, chymotrypsin C, proteasome subunit beta type-2, and cathepsin L1, chymotrypsin C, proteasome subunit alpha type 4. Reducing or increasing the number of proteases in the combinations did not further increase the prediction accuracy (Fig 2C).

Additionally, ROC curves were plotted for the three predictor proteins elastase 3A, cathepsin L1 and proteasome subunit alpha type-4 (Fig 2D). The area under the curve (AUC) of the ROC plots indicates how well the proteases correctly classify the individual supernatant samples as HC or IBS. A perfect classification would correspond to an AUC of 1 whereas 0.5 would be considered random classification. The AUC values for the single proteases were not lower than 0.86. However, the combination of the three proteins revealed with 0.98 the highest AUC value (Fig 2D).

It is of note that one of the most specific proteins to differentiate IBS from UC and HC samples, complement C3, is not included in the most accurate triple-protease markers derived by linear discriminant analysis. The key idea of the linear discriminant analysis (and, generally, approaches for identifying multi-marker panels) is to find combinations of biomarkers where each biomarker contributes orthogonal information to improve the prediction power of the classificator. Hence, it appears that the single protease biomarker, complement C3, is outperformed in terms of prediction accuracy by a multi-marker panel, capturing more relevant biological variance (and covariates) than a single marker alone. Nevertheless, complement C3 is still a very good marker having a prediction accuracy of 92.5% in the combination with chymotrypsin C (best when two proteases were combined) and 89% in the triple combination with cathepsin C and proteasome subunit alpha type 4 with a (8th best prediction accuracy).

### Functional links between protease profile and neural effects of supernatants

We evaluated a possible association between protease abundance (log_10_ ratio) in IBS supernatants and the neuronal activation, evoked by supernatants. As the PAR1 antagonist inhibited the neuronal activation evoked by IBS but not UC supernatants, we focused on those proteases which were significantly more abundant in IBS supernatants and correlated their abundance with the SCH79797 induced percentage decrease in supernatant induced nerve activation (neuroindex). The assumption was that the higher the abundance of proteases in the supernatants and their contribution to the nerve activation the stronger the inhibitory effect of SCH79797 on supernatant induced nerve activation. Indeed, we found that, except for chymotrypsin C, the abundance of proteases significantly correlated with the efficacy of SCH79797 to inhibit nerve activation (Table 2).

**Table 2:** Correlation between proteases upregulated in IBS supernatants and the PAR1 component of neural activation as determined by the relative change in SCH79797 induced decrease in neuroindex.

Protease	Correlation coefficient	P value
Complement C 3	-0.690	0.004
Complement C 3 (unspecified isoform)	-0.733	0.001
Elastase 3A	-0.637	0.009
Plasminogen	-0.520	0.044
Chymotrypsin-C	-0.475	0.071
Proteasome beta subunit type-2	-0.543	0.034

Correlation coefficient and *P* values from Pearson correlation analysis.

**Fig 3:**
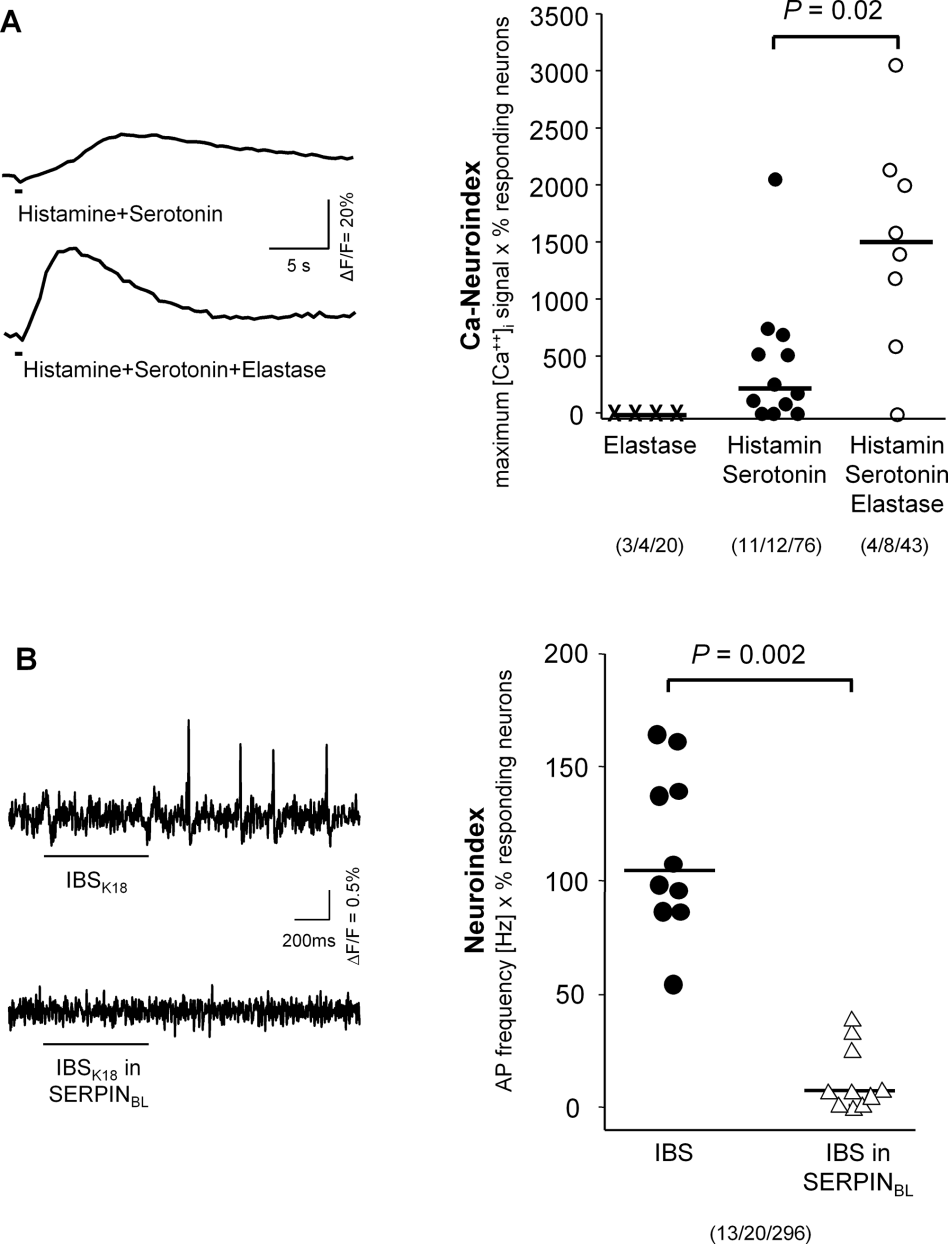
Role of elastase in the activation of submucous neurons. (A) Traces show the synergistic effect of adding 100nM elastase to a mediator mix containing 1μM histamine and 1μM serotonin. The right panel displays the analysis of several such experiments. Elastase alone had no effect. The mix of histamine and serotonin induced a nerve activation indicated by an increased Ca-neuroindex (product of maximum [Ca^2+^]_i_ signal x % responding neurons, calculated for each ganglion). Addition of elastase significantly enhanced the [Ca^2+^]_i_ neuroindex (Mann-Whitney Rank Sum Test, tissue/ganglion/neuron). (B) Inhibitory effect of 15nM SERPIN_BL_ from *Bifidobacterium longum* on IBS supernatant evoked spike discharge in human submucous neurons (left panel). The neuroindex (product of spike frequency and % of responding neurons) evoked by IBS supernatants from 10 patients was significantly reduced (Wilcoxon signed Rank Test for paired data; each symbol represents one patient sample; right panel). Bars below the traces indicate the duration of application. Numbers in parentheses indicate number of tissues/ganglia/neurons studied.

We additionally performed two sets of experiments to emphasize the importance of proteases for nerve activation by IBS supernatants. We first studied the functional role of elastase. Elastase (up to 1μM) had no direct effect on human submucous neurons. However, elastase potentiated the neuronal activation evoked by the mediator mix of histamine and serotonin (both 1 μM); the Ca^++^-neuroindex increased from 216 (21/651) to 1499 (746/2111) (*P* = 0.02, Fig 3B). We recently demonstrated the same effect for tryptase [7]. Second, we incubated the IBS supernatants with 15nM SERPIN_BL,_ which is known to specifically inhibit elastase-like proteases [20]. For these experiments, we used guinea pig preparations, because they allowed us the most efficiently use the limited supply of SERPIN_BL_. SERPINBL prevented the nerve activation induced by IBS supernatants (Fig 3C).

## Discussion

This study identified proteases and in particular those involving PAR1 signaling pathways as markers to distinguish IBS from HC and UC in remission. Further studies should now evaluate the potential of proteases acting through PAR1 as biomarkers in a clinical setting.

We acknowledge that our findings are based on relatively low number of patient samples and therefore need validation at higher scales. Additionally, most IBS samples came from patients with diarrhea and we cannot entirely rule out that other IBS phenotypes associated with constipation show additional differences in protease expression patterns. In our mind this does not compromise the validity of our results as our previous work showed that nerve activation by IBS supernatants occurred independent of IBS subtypes [3]. Our results stress the relevance of proteases signaling through PAR1 in IBS but not UC. This does not rule out that some proteases in the IBS supernatant signal through other PARs in particular PAR2. Part of the PAR1 dominance in our study is explained by the finding that proteases signal in human enteric neurons almost exclusively via PAR1 [8,9]. This is not the case in rodent models in which neural PAR2 are functionally expressed on enteric neurons and thereby contribute to nerve activation [9,24]. In the human intestine PAR2 has a small pro-secretory effect by directly activating epithelial cells [9].

Supernatants from UC and IBS activated enteric neurons. We previously demonstrated the dominant role of proteases in nerve activation as the serine protease inhibitor FUT-175 abolished responses to IBS supernatants [3,6]. This was also the case for UC supernatants based on the finding that FUT-175 also decreased spike discharge in response to UC supernatants (this study). However, the pharmacology of the response was strikingly different. While nerve activation of IBS supernatants was prevented by PAR1 antagonism, PAR1 played no role in nerve activation by UC supernatants. It was beyond the scope of our study to investigate involvement of other PARs or PAR-independent pathways. The rationale to also test UC supernatants was to include patients with similar symptoms that were clearly related to immune activation. Findings with fecal samples support differential expression of proteases in IBS and UC. While fecal proteases derived from IBS patients increased, those from UC patients decreased visceral sensitivity in mice [25].

This study focused on proteases, which are the main neuroactive component in mucosal biopsy supernatants from IBS [3,6] and UC patients (this study). It needs to be mentioned that a number of other proteins are up or downregulated in IBS supernatants compared to healthy controls. Many of these are part of signaling cascades involved in host defense and inflammatory processes (see Table 1 and S2 Table) which agrees with the concept that immune activation is one prominent feature of IBS [1].

The patients were not asked to stop their medication unless 24 hours before endoscopy. We therefore cannot rule out that the maintenance medication taken by the patients with quiescent UC influenced the protease profile in UC supernatants. Having medical treatment for inflammatory bowel disease does not alter protein expression since cytokine expression in mucosal biopsy supernatants from UC patients differed significantly between inflamed and adjacent non-inflamed regions despite the medications taken by the patients [26].

We identified numerous proteins which were up or downregulated in IBS biopsy supernatants compared to UC or HC supporting the view that IBS and UC are separate entities despite some common symptoms. Among the upregulated proteins were 17 proteases. The rational to focus on proteases was our previous finding that proteases mediate nerve activation of IBS supernatants [3,6] and synergistically potentiate the responses to the non-protease neuroactive substances present in the supernatants [7]. Our current study also demonstrated the important role of proteases for UC supernatant evoked nerve activation. A recent study found increased expression of trypsin 3, a protease released from epithelial cells, in mucosal biopsies from IBS patients [27]. Trypsin-3 application activated sensory and submucous neurons. We were able to measure trypsin 1, but did not detect trypsin 2 and 3 in our study. Trypsin 1 levels were not significantly different between sample groups. Evidence for enhanced trypsin-like activity in our study was the higher abundance of proteasome subunit type beta-2 in IBS supernatants. Proteasomes are involved in cellular protein quality control, help to degrade proteins and are involved in inflammatory processes by regulating expression of cytokines. Proteasomes with trypsin-like activity were also upregulated in mucosal biopsies from IBS patients and this was associated with the degradation of the tight junction protein occludin and increased mucosal TNFalpha [28].

There are a number of studies emphasizing the role of proteases as neuroactive components in IBS biopsy supernatants [3,5,24,27]. IBS patients receiving a 6 weeks treatment with the probiotic strain *Bifidobacterium longum NCC3001* reported an improved overall symptom score independent of whether they had diarrhea or mixed stool pattern [29]. The treatment also caused reduced responses to negative emotional stimuli in multiple brain areas, suggesting that some soluble factor directly or indirectly released during *Bifidobacterium longum* NCC3001 acted systemically. In our study the SERPIN from *Bifidobacterium longum* prevented activation of enteric neurons by IBS supernatants.

Proteases are released from a number of sources in the gut of IBS or UC, including nerves, epithelial cells, inflammatory cells, pancreas or microbiota [11]. The increased levels of elastolytic proteases found in the IBS supernatants were likely a consequence of a locally restricted upregulation because fecal elastase levels were increased in only 13% of IBS patients [30]. Elastase 3a, although considered a pancreatic enzyme, is expressed throughout the human intestine including the colon [22] (see also proteomicsdb.org). The substrates of cathepsin L1, another protease highly abundant in IBS supernatants, include elastin, which is also, yet to a lesser extent, cleaved by elastase 3a. Elastin and collagen determine the mechanical properties of connective tissue. Interestingly, the joint hypermobility syndrome, which is a connective tissue disorder, was significantly associated with functional gastrointestinal disorders including IBS [31].

In summary, we demonstrated that nerve activation by mucosal biopsy supernatants depends on proteases. This is a common feature of UC and IBS and may relate to some common gastrointestinal symptoms. However, only proteases in IBS supernatants signal through PAR1. Proteomics reveal an IBS characteristic protein pattern and in particular a characteristic protease profile. Proteases such as trypsin and elastase activate enteric neurons mimicking the effect of supernatants. A serine protease inhibitor from a probiotic *Bifidobacterium longum* strain prevented the IBS supernatant induced nerve activation. Taking together our findings identified proteases profiling as a promising strategy to develop IBS biomarkers.

## Supporting information

S1 Table Characteristics of patient samples for generation of supernatants. (DOCX)

S2 Table Quantified proteins in mucosal biopsy supernatants. (XLSX)
