# Supplementary material for: Protease signaling through protease activated receptor 1 mediate nerve activation by mucosal supernatants from irritable bowel syndrome but not from ulcerative colitis patients
Source: PLoS One. 2018 Mar 12;13(3):e0193943. doi: 10.1371/journal.pone.0193943 (PMC5846775; doi:10.1371/journal.pone.0193943)
Supplement: S1 Table — (DOCX) [file pone.0193943.s001.docx]

| **Healthy controls (HC)^+^** | **Age/**  **Gender** | **Neuroimaging: No of human tissues/ganglia/neurons** | **Proteomeanalysis** |
| --- | --- | --- | --- |
| *^#^HC_B3_ | 30/m | 1/2/11 | yes |
| *^#^HC_B5_ | 22/f | 5/8/59 | yes |
| *^#^HC_B177_ | 61/f | 2/2/15 | yes |
| *^#^HC_B178_ | 55/m | 2/2/14 | yes |
| ^&^HC_A6_ | 20/f | 1/3/18 | yes |
| ^&^HC_A8_ | 21/f | 4/6/51 | yes |
| ^&^HC_A16_ | 28/m | 4/8/67 | yes |
| **Ulcerative colitis in remission (UC)** | **Age/**  **Gender/** | **Neuroimaging: No of human tissues/ganglia/neurons** | **Proteome analysis** |
| UC_A1_ MT:1,2 | 55/m | 3/3/36 | - |
| UC_A23_ MT:3 | 64/f | 2/2/24 | - |
| UC_A10_ MT:2,3,4 | 42/f | 2/2/17 | - |
| UC_A12_ MT:3 | 54/f | 2/2/25 | - |
| UC_A13_ MT:3,4,5 | 60/f | 2/2/19 | yes |
| UC_A15_ MT:3 | 62/m | 5/9/48 | yes |
| UC_A17_ MT:3,6 | 34/f | 3/2/20 | yes |
| UC_A21_ MT:3,4,5 | 45/f | 4/5/39 | yes |
| UC_A23_ MT:3,3 | 45/m | 3/3/37 | yes |
| UC_A25_ MT:3,4,7 | 49/f | 3/3/23 | yes |
| UC_A4_ MT:n.k | 19/m | 3/4/27 | yes |
| UC_V110_ MT:n.k | 21/f | 1/1/6 | - |

**S1 Table.** **Characteristics of patient samples for generation of supernatants**

| **Symptomatic**  **IBS (IBS)** | **Age/Gender/Phenotype** | **Neuroimaging: No human (Hu) or guinea pig (Gp) tissues/ganglia/neurons** | **Proteome analysis** |
| --- | --- | --- | --- |
| IBS_A58_ | 21/f/IBS-A | Hu: 4/4/21 | yes |
| IBS_A46_ | 31/m/IBS-C | Hu: 2/2/21 | - |
| IBS_A40_ | 35/f/IBS-A | Hu: 2/2/21 | - |
| IBS_K7_ | 28/f/IBS-D | Hu: 2/2/14, Gp: 2/2/40 | yes |
| IBS_K9_ | 49/m/IBS-D | Hu: 4/4/22, Gp: 2/2/28 | yes |
| IBS_K5_ | 37/f/IBS-D | Hu: 3/3/37, Gp: 1/2/24 | - |
| IBS_K11_ | 44/f/IBS-D | Hu: 3/3/21, Gp: 1/2/29 | - |
| IBS_K14_ | 39/f/IBS-D | Gp: 1/2/34 | - |
| IBS_K15_ | 53/f/IBS-D | Gp: 1/2/25 | - |
| IBS_K17_ | 40/f/IBS-D | Gp: 1/2/25 | - |
| IBS_K18_ | 67/f/IBS-D | Gp: 1/2/29 | - |
| IBS_K21_ | 31/m/IBS-D | Gp: 1/2/25 | - |
| IBS_K27_ | 60/f/IBS-D | Gp: 2/2/37 | - |
| *IBS_B137_ | 35/f/IBS-D | Hu: 4/4/22 | yes |
| *IBS_B134_ | 32/m/IBS-D | Hu: 4/4/19 | yes |
| *IBS_B24_ | 68/f/IBS-D | Hu: 3/3/14 | yes |
| *IBS_B141_ | 35/f/IBS-C | Hu: 2/3/16 | - |
| IBS_B146_ | 32/m/IBS-D | Hu: 1/1/16 | - |
| IBS_V22_ | 37f/IBS-D | Hu: 2/2/11 | yes |
| IBS_V69_ | 17/f/IBS-D | Hu: 2/2/9 | yes |

^+^ subscripted letters indicate origin of supernatants: A = Amsterdam, B = Bologna, K = Krefeld, V = Vilsbiburg; * samples from these controls and patients have also been used in a previous study (Buhner et al., 2009); ^#^ recruited by public advertisement;^&^ samples taken during preventive endoscopic checkup.

MT = Maintenance therapy: 1 Budenosid; 2 Ursodesoxycholic acid; 3 Mesalazin; 4 Azathioprin; 5 Prednisolon; 7 Infliximab; n.k. not known
